# Neutralization heterogeneity of circulating SARS-CoV-2 variants to sera elicited by a vaccinee or convalescent

**DOI:** 10.2217/fvl-2021-0100

**Published:** 2022-04-26

**Authors:** Meng Zhang, Yixin Gong, Shunchang Jiao

**Affiliations:** ^1^Department of Research & Development, Beijing DCTY^®^ Biotech Co., Ltd, 86 Shuangying West Road, Beijing, 102200, People's Republic of China; ^2^Department of Oncology, Chinese PLA General Hospital, 28 Fuxing Road, Haidian, Beijing, 100853, People's Republic of China

**Keywords:** convalescent, COVID-19, neutralization, vaccine, VOCs

## Abstract

COVID-19, which was first reported in December 2019 in China, has caused a global outbreak. Five variants of concern (VOCs) have been identified in different countries since the global pandemic, namely, Alpha (B.1.1.7), Beta (B.1.351), Gamma (P.1), Delta (B.1.617.2) and Omicron (B.1.529). Although multiple vaccines have been found to be effective, some of the amino acid changes may increase the infectivity of virus and decrease the sensitivity to antibodies. Here we characterize the VOCs and discuss their sensitivity to antibodies elicited by convalescent and vaccinee sera. In conclusion, several variants display a reduction in the susceptibility to neutralization antibodies generated by natural infection or vaccination, which threatens the containment of the epidemic.

SARS-CoV-2 is a novel β-coronavirus causing COVID-19 [1,2]. It rapidly became a pandemic since first reported in China in December 2019 [3]. Over 404 million confirmed cases and more than 5 million deaths have been identified as of 12 February 2022. Coronaviruses have genetic proofreading mechanisms [4,5] and RNA viruses are known to have higher mutation rates than DNA viruses [6,7]. Although SARS-CoV-2 shows evidence of some seasonal waning [8], the emergence of variants under natural and environmental selective pressure increases the risk of the spread of SARS-CoV-2. Five major variants of concern (VOCs) have been reported: Alpha (B.1.1.7) emerged first in the UK [9], Beta (B.1.351) in South Africa [10], Gamma (P.1) in Brazil [11], Delta (B.1.617.2) in India [12] and Omicron (B.1.529) [12] in South Africa.

SARS-CoV-2 encodes at least 29 proteins in its (+) RNA genome, among the four structure proteins (the spike [S], membrane [M], envelope [E] and nucleocapsid [N] proteins), with S and N proteins representing the most distinctive features of the virus [13,14]. SARS-CoV-2 interacts with human angiotensin-converting enzyme 2 (hACE2) for cell entry [3], and the spikes of SARS-CoV-2 have unusual freedom on the viral envelope, allowing it to better engage with the cellular receptor, ACE2 [14]. The spike protein consists of S1 and S2 subunits, and it is active when cleaved by proteases, which is important for the receptor recognition and membrane fusion [15,16]. S1 can be further divided into an N-terminal domain (NTD) and a C-terminal domain (CTD), both of which can function as a receptor-binding entity. The S1 CTD was identified as the key region of SARS-CoV-2 that interacts with the hACE2 receptor [16]. Most of the neutralizing antibodies and vaccines are designed based on the spike protein sequence, especially the receptor-binding domain (RBD) [17,18]. The RBD-targeting antibodies generally seem to exhibit higher neutralization activity to several VOCs, although the NTD-targeting antibody also shows high neutralization activity to wild-type (WT) SARS-CoV-2 [19]. The Alpha variant is sensitive to most RBD-directed antibodies, whereas both Alpha and Beta are markedly resistant to neutralization by NTD-directed antibodies [20]. In addition, it has been reported that the neutralization activity of NTD-targeting antibodies against virus carrying E484K and other mutations in the NTD was abolished [21]. However, RBD-targeting antibodies may induce resistance in VOCs; thus, the combination of non-RBD- and RBD-targeting antibodies could be used as a therapeutic cocktail for the VOC [19,20].

Among coronaviruses, point mutations have been demonstrated to confer resistance to neutralizing antibodies in MERS-CoV [22] and SARS-CoV [23,24]. Thus, single amino acid changes are worth monitoring because they can be phenotypically and functionally relevant, and mutations in the spike protein and other proteins have been detected in several SARS-CoV-2 variants. The first identified SARS-CoV-2 mutation is D614G. It is caused by an A-to-G nucleotide change in the spike protein in WT [25]. The earliest examples of sequences carrying the D614G were found in China and Germany in late January 2020. By early April 2020, G614 has become the dominant form in the pandemic, and this is facilitated by the higher transmissibility of D614G.

The Ct (the cycle threshold) is used as a surrogate for relative viral loads; lower Ct values indicate higher viral loads. The association of the G614 variant with low Ct values *in vivo* has been reported [25–27], and G614-bearing viruses had significantly higher infectious titers (2.6- to 9.3-fold increase) than their D614 counterparts [25]. G614 also seemed to increase the spike stability and membrane incorporation [28] and reduce S1 subunit shedding from virions [28–30], which is associated with increased infectivity but not with increased mortality [31]. Studies on hamsters demonstrated that the G614 variant is as sensitive to the serum specimens as the D614 strain [32], and convalescent sera exhibited equivalent or better neutralization of G614-bearing pseudoviruses compared with D614-bearing ones. This suggests that G614-bearing virions are not intrinsically more resistant to neutralization by convalescent sera [25,33].

In this review, we sketched the key mutations and resistance to antibodies of the VOCs that pose a public health challenge during the COVID-19 pandemic.

## Variants of concern

On 26 November 2021, five SARS-CoV-2 variants have been designated as VOCs. The emergence of VOCs poses a serious threat to the global public health as they harbor multiple mutations that cause the increasing transmission and partly decreasing susceptibility to neutralization antibodies elicited by convalescents and vaccinees.

### Alpha

Alpha is the first SARS-CoV-2 VOC that emerged in the UK in September 2020; later, it was detected in multiple countries worldwide [34]. This variant presents 23 nucleotide mutations (14 nonsynonymous mutations, 6 synonymous mutations and 3 deletions). Multiple mutations encoding for the spike protein are of most concern, such as the deletion 69–70, deletion 144, N501Y, A570D, P681H, D614G, T716I, S982A and D1118H (Table 1) [9]. Alpha is 56% more transmissible than pre-existing variants of SARS-CoV-2 [35]. One of the mechanisms accounting for increased transmissibility is enhanced spike protein-binding affinity for the ACE2 receptor. The RBD of Alpha bound ACE2 with 1.98-fold greater affinity than the WT SARS-CoV-2 RBD (Kd 203.7 + 57.1 nM vs 402.5 + 112.1 nM) [36]. The N501Y mutation situated in the RBD has been shown to enhance binding affinity to the host cell ACE2 receptor [37,38]. The P681H mutation located immediately adjacent to the furin cleavage site in the spike protein is important for infection and transmission [39,40]. The deletion at positions 69 and 70 of the spike protein is linked to immune evasion in immunocompromised patients and associated with diagnostic test failure for the probe targeting of the spike protein [41]. Moreover, it has been demonstrated that the Alpha variant increases the risk of mortality compared with pre-existing variants [42,43].

**Table 1.** Non-synonymous mutations and deletions in variants of concern.

	Alpha (B.1.1.7)	Beta (B.1.351)	Gamma (P.1)	Delta (B.1.617.2)	Omicron (B.1.529)	
ORF lab	T1001I	T265I	S1188L	P314L	K856R	P314L
	A1708D	K1655N	K1795Q	G662S	S2083 del	I1566V
	I2230T	H2799Y		P1000L	L2084I	
		S2900L			A2710T	
		K3353R			T3255I	
	SGF 3675–3677 del		SGF 3675–3677 del		P3395H	
		D4527Y	E5665D		LSG3674–3676 del	
		T59121			I3758V	
S	HV69–70 del			T19R	A67V	G496S
		L18F	L18F	E156 del	HV69–70 del	Q498R
		D80A	T20N	F157 del	T95I	N501Y
	Y144 del	D215G	P26S	R158G	GVY142–144 del	Y505H
		K417N	K417T	K417N	K417N	T547K
		E484K	E484K	E484K	N211 del	D614G
	N501Y	N501Y	N501Y	T478K	G339D	H655Y
	D614G	D614G	D614G	D614G	S371L	N679K
	A570D	R246I	D138Y	D950N	S373P	P681H
	P681H		R190S	P681R	S375F	N764K
		A701V		L452R	Y145D N440K	D796Y
	T716I	LLA241–243 del	H655Y		G446S	Q954H
	S982A		T1027I		S477N	N856K
	D1118H				E484A	Q954H
					Q493R	N969K
					T478K	L981F
Orf3a		Q57H		S26L		
Orf8	Q27 stop	S171L	E92K	D119 del		
	R52I		Ins28269–28273	F120 del		
	Y73C					
E		P71L			T9I	
M				I82T	D3G	
					Q19E	
					A63T	
N	D3L	T205I	P80R	D63G		
	S235F			R203M		
				D377Y		

### Beta

In December 2020 a new SARS-CoV-2 variant, namely, Beta, emerged and spread throughout South Africa. This new strain presents 19 mutations. In addition to D614G, eight other mutations are situated in the spike protein. The N501Y, E484K and K417N mutations are at key residues in the RBD, L18F, D80A and D215G located in the NTD, A701V in loop2 (Table 1) [10]. The N501Y mutation recently identified in Alpha emerged in the UK, it demonstrated the potential to enhance the binding affinity to hACE2 [37,38]. The uncommon mutation E484K has been shown to modestly enhance the binding affinity of the ACE2 receptor [38]. Both E484K and N501Y located at the receptor-binding motif (RBM), which is important for the binding with hACE2. The K417N mutation has a negligible impact on the binding affinity to hACE2 [38], although the binding affinity of SARS-CoV-2 to hACE2 is also associated with K417 situated in the spike RBD region [10,44,45]. The Beta RBD bound ACE2 at 4.62-fold greater affinity than WT SARS-CoV-2 RBD (Kd 87.6 + 25.5 nM vs 402.5 + 112.1 nM) [36]. The above analysis revealed that Beta has been estimated to be 50% more transmissible than pre-existing variants in South Africa [46].

### Gamma

In December 2020, another new variant was detected in Manaus (Amazonas state, north Brazil), named Gamma, and is associated with a case of reinfection [11,47]. Gamma has 17 unique amino acid changes, 3 deletions, 4 synonymous mutations and 1 four-nucleotide insertion (Table 1) [11]. The E484K and N501Y mutations situated in the RBD detected in the Beta strain are also present in Gamma, which are associated with increased binding affinity to hACE2 and infectivity [38]. In addition, Gamma and Beta share another mutation in the spike protein (K417N/T), which is also situated in the RBD of S protein [10] and contributes to the enhanced binding affinity of SARS-CoV-2 to hACE2 [44,45]. Furthermore, Gamma, Alpha, and Beta share the ORF1b deletion [11]. The mutations shared between Gamma, Alpha, and Beta seem to be associated with increased transmissibility. Although there is no evidence that Gamma causes more severe symptoms or higher mortality, cases of reinfection have been reported. It is important to rapidly investigate whether the new variant leads to reinfection in previously exposed individuals as well as to detect the possible emergence of new variants in the near future. Furthermore, it is notable that the variants Alpha, Beta, and Gamma share an N501Y mutation, efficiently infecting cells carrying the ACE2 orthologs of rats, mice and human, whereas the WT strain only infect cells expressing hACE2, the N501Y mutation lead to the increase of the intermediate hosts and the potential risk of the spread of SARS-CoV-2 [48].

### Delta

The Delta variant was first detected in October 2020 in India [12]. It presents multiple mutations situated in the spike protein, including T19R, E156, and F157 deletion, R158G, L452R, T478K, E484K, D614G, P681R and D950N (Table 1). Furthermore, some delta variants acquired an additional K417N mutation that has been reported to increase the binding affinity of SARS-CoV-2 to hACE2 [44,45]. Moreover, L452R was found to significantly increase the free energy of the RBD–ACE2 binding complex and was predicted to cause a much higher binding affinity to the receptor and increased infectivity [49,50]. The mutations in the RBD of the spike protein may reduce susceptibility to antibodies elicited by sera of prior infection and vaccination. The L452R and P681 mutations situated in the RBD were found to be responsible for the resistance to certain monoclonal antibodies [51,52]. In addition, the Delta variant is much more contagious owing to its higher replication rate and virus titers upon early infection [53,54]. The faster replication of the virus also poses challenges to the durability of COVID-19 vaccines.

### Omicron

Omicron was first reported in South Africa and was designated as VOC in November 2021 [12]. The new variant harbors the largest number of mutations among the five VOCs, with 37 mutations in the spike and more than 20 mutations outside the spike (Table 1). It is noteworthy that Omicron shares important mutations in the spike with the other four VOCs, including N501Y, K417N, P681H and E484K/A [9,10]. The emergence of Omicron increased the risk of breakthrough infections as it has the potential for immune evasion. Breakthrough infections mean that individuals who have received vaccines are infected with the SARS-CoV-2 variant. Omicron breakthrough infections have been reported in individuals who received three doses of mRNA vaccines [55].

Taken together, with the persistence of the SARS-CoV-2 pandemic, several SARS-CoV-2 variants have been identified. These variants with multiple shared mutations in the spike protein are potentially associated with an increase in transmissibility and disease severity or propensity for reinfection of individuals. Thus, it is critical to monitor the new emergence mutations and assess the efficacy of existing vaccines or antibodies used for the prevention or treatment of COVID-19.

## Neutralization heterogeneity of VOCs to sera elicited by vaccines or previous infections

In response to the urgent need to develop effective vaccines against SARS-CoV-2, numerous vaccine development technologies have been explored. Multiple vaccines against SARS-CoV-2 have been approved by different countries for clinical use, for example, two mRNA vaccines (BNT162b2 from Pfizer-BioNTech, USA; mRNA-1273 from Moderna, USA) [56,57], three inactivated vaccines (BBIBP-CorV from Sinopharm, China; CoronaVac from Sinovac, China) [58–60], and four adenoviral vectored vaccines (Sputnik V, Russia; AZD1222 from AstraZeneca-Oxford, UK; JNJ78436735 from Johnson & Johnson, USA; Ad5-nCoV, China) [61–63].

The trimeric S protein mediates host cell binding and entry, and the design of the major targets of neutralizing antibodies and vaccines is based on the spike protein sequence of the first isolated virus (Wuhan-Hu-1; GenBank accession no. NC_045512) [17,18,64]. However, mutations that occurred within the spike RBD region might lead to the decrease in the neutralization effect of antibodies elicited by convalescent sera and vaccines designed according to the spike sequence of early SARS-CoV-2 strains, posing additional challenges in controlling the pandemic. The aforementioned lineages are each characterized by numerous mutations in the spike protein, raising concerns of whether they can escape from therapeutic antibodies and vaccine-induced sera. Here, we elaborated the neutralization heterogeneity of SARS-CoV-2 variants to antibodies elicited by convalescents and vaccinees (Table 2).

**Table 2.** Efficacy of SARS-CoV-2 vaccines and neutralization activity to the variants.

Vaccine	Efficacy at preventing infection (%)	Fold reduction	Efficacy at preventing infection (%)	Fold reduction	Efficacy at preventing infection (%)	Fold reduction	Efficacy at preventing infection (%)	Fold reduction	Efficacy at preventing infection (%)	Fold reduction	Ref.
	WT	Alpha		Beta		Gamma		Delta		Omicron	
BNT162b2 (Pfizer-BioNtech)	95.0	2.1	93.7	6.5	75.0	2.2–6.7	82.0	2.5–3.3	88.0	44	[20,57,74,78–80,83,88,91]
mRNA-1273 (Moderna)	94.1	2.3	99.2	8.6	96.4	2.8–4.5	85.0	3.0–3.2	73.1	33	[20,56,79,80,83,88,91]
AZD1222 (AstraZeneca)	66.7	9.0	74.6	NA	10.4	NA	31.0	2.6	49.0	36	[62,81,83,89–91]
NVX-CoV2373 (Novavax)	89.3	2.0	85.0	NA	50.0	NA	43.0	NA	NA	NA	[67,71,91]
BBIBP-CorV (Sinopharm)	79.0	0.4	NA	0.6	NA	NA	41.0	NA	NA	NA	[33,83,91]
CoronaVac (Sinovac)	50.7	0.5	NA	0.7	NA	0.3	28.0	2.5	NA	NA	[33,76,91]

NA: Not available; WT: Wild-type.

Convalescent sera harbored similar neutralization activity against Alpha when compared with the D614G strain [20,65–67]. Furthermore, the sera elicited by Pfizer BNT162b2 and Moderna mRNA-1273 vaccines also maintain effective neutralization activity against the Alpha variant [20,31,65–70]. The Novavax COVID vaccine was more than 85% effective against Alpha lineage [71]. The Alpha variant showed little resistance to the neutralizing activity of the BBIBP-CorV and the CoronaVac vaccinee sera [59]. However, Alpha is refractory to neutralization by most antibodies to the NTD of the spike protein and relatively resistant to a few monoclonal antibodies to RBD [20,48,67].

Beta is more worrisome when compared with Alpha in that it is not only refractory to neutralization by most NTD antibodies and multiple RBD antibodies [20,48] but also exhibits varying degrees of resistance to convalescent serum and vaccine-elicited antibodies [20,31,65,66,70,72,73]. Antibodies elicited by BNT162b2 neutralized pseudotyped virus carrying the Beta spike protein with a 3.1-fold reduction in IC50 compared with D614G [58]. The Novavax COVID vaccine is less than 50% effective against the Beta strain [71]. Neutralizing antibody titers to Beta reduced 3.5-fold from recipients of mRNA-1273 as compared with D614G [68]. The neutralization activity of the BBIBP-CorV and CoronaVac vaccinee sera against Beta also significantly decreased [33]. In addition, it has been reported that 7 days after the booster vaccination, neutralizing activity against Beta (median neutralizing titer of 1:5) significantly reduced in comparison with the D614G mutation (1:160) [65]. This indicates the necessity of vaccination even in people who had recovered fromCOVID-19 to avoid reinfection with the SARS-CoV-2 variants.

Beta and Gamma share the same mutations in the RBD region, except for the K417N mutation is situated in the RBD of Beta and Gamma RBD adopted a K417T mutation, thus it's reasonable that Gamma is also highly resistant to therapeutic antibodies [10,11,38] and sera from convalescent and vaccinee. It has recently been demonstrated that the Gamma lineage is also resistant to multiple monoclonal antibodies, including monoclonal antibodies targeting RBM and NTD [74]. In addition, the neutralization activity of antibodies from the sera of convalescents and vaccinees to Gamma is also reduced, although the magnitude of the loss is modest as compared with that for Beta [20,74–76].

Delta also exhibits resistance to antibodies elicited by vaccinee and convalescent sera. It has a higher replication rate and the ability to evade immunity, which contribute to the rapid spread of the variant. Based on the real-world data, vaccines remain protective against the variant, although multiple vaccines seemed to reduce the effectiveness against Delta [77]. The effectiveness of BNT162b2 and mRNA-1273 to persons infected with Delta is 88 and 67%, respectively [78]. Furthermore, the neutralization antibody from vaccines (BNT162b2, mRNA-1273 or AZD1222) and convalescent sera exhibited less neutralizing activity against Delta, and the waning immunity may be responsible for the declined effectiveness and increased risk of reinfection [77,79–81].

Although it may not cause severe disease or death, Omicron evades neutralization antibodies from convalescents and vaccinees. In addition, the reduced effectiveness of vaccines against Omicron has been reported, and the effectiveness of BNT162b2 reduced to 70% [82]. The neutralization activity of BNT162b2, mRNA-1273, and AZD1222 against Omicron decreased by 44-, 33- and 36-fold, respectively [83]. However, booster vaccines may increase the neutralization antibody titers and reduce the risk of breakthrough infection [84].

Furthermore, studies have demonstrated that the neutralization antibody titers decreased 6 months after receiving the second dose of the vaccines [85]; thus, booster vaccination is needed to enhance the waning immunity and prevent virus or breakthrough infection. Homologous and heterologous prime-boost vaccination strategies have been approved, and their effectiveness and safety have been evaluated. To overcome the ongoing pandemic and the shortage of vaccines worldwide, the safety and efficacy of heterologous prime-boost vaccination strategies have been studied. Heterologous prime-boost vaccination means two doses of vaccine used to prevent COVID-19 are different. The first and second doses of vaccine may be from different platforms, for example, ChAdOx1 for the first dose and mRNA-1273 or BNT162b2 for the booster dose. Heterologous prime-boost vaccination regimes could induce a more robust humoral immunity, and only mild adverse events were reported [77,86,87]. Higher neutralization antibody titers were elicited after heterologous prime-boost vaccination. Homologous boost vaccines were found to increase the neutralization titers by 4.2- to 20-fold after administration, whereas heterologous prime-boost vaccines increased by 6.2- to 76-fold) [86]. Heterologous prime-boost vaccination may be an excellent strategy to control the SARS-CoV-2 pandemic.

## Conclusion

The emergence of SARS-CoV-2 in December 2019 posed a significant threat to global health. Five VOCs subsequently emerged and widely spread during the pandemic. Alpha was the first detected variant, and it demonstrated increased transmissibility and mortality. Beta shares several mutations with Alpha and also presents a higher transmissibility compared with WT. Gamma was subsequently detected in December 2020 in Brazil. It is also more infectious and associated with reinfection. The emergence of Delta caused resurgence of SARS-CoV-2 outbreak. The increased transmission of Delta is related to the higher replication rate. Omicron has recently emerged in multiple countries, which harbors a high number of mutations in the spike protein and may also be associated with higher transmissibility.

Vaccines are effective against infection with the original SARS-CoV-2, however, their effectiveness against several VOCs is reduced. The emergence of VOCs harbored multiple mutations in the spike protein could escape the neutralizing antibody response. Sera obtained from vaccinees exhibited slightly reduced but largely preserved activity against Alpha. The Beta variant is more worrisome as it is less sensitive or even insensitive to a large part of the vaccinee sera tested. Gamma shares three mutations in RBD with Beta, namely, E484K, K417N/T, and N501Y, and also has significantly decreased neutralization activity even in fully vaccinated individuals. Delta also exhibits resistance to antibodies elicited by vaccinees and convalescents. In addition, Gamma and Delta may increase the risk of reinfection. Omicron also has the potential to evade immunity as it shares several mutations of concern with other VOCs. The waning immunity is another important reason for the resurgence of epidemic, and booster vaccines could induce a strong immune response without causing serious adverse reactions. Heterologous prime-boost vaccination strategies have been employed to deal with the waning immunity.

## Future perspective

We speculate that SARS-CoV-2 will not be eliminated in the next few years and will even coexist with people for a long time as the virus may continue to evolve. It is possible that many more serious variants that have the ability to evade immunity will emerge worldwide over time. Genetic surveillance is necessary for the early identification of newly emerging variants. Furthermore, resistance to antibodies, faster replication, and waning immunity pose challenge to vaccines. We could not rely on vaccines alone to avoid infection; measures such as wearing of mask, social distancing, frequent handwashing and ventilation improvement should be taken to reduce the spread of SARS-CoV-2. Meanwhile, next-generation vaccines against key mutations should also be fabricated to avoid a new wave. We believe that the epidemic will be controlled through global cooperation.

**Executive summary**

**Variants of concern**

- Five SARS-CoV-2 variants have been currently designated by the WHO as variants of concern (VOCs), namely, Alpha, Beta, Gamma, Delta and Omicron.
- Alpha is the first VOC that emerged, and it increased the risk of mortality. The N501Y and P681H mutations in the spike protein increased the transmissibility of the virus.
- Beta shares the D614G and N501Y mutations with Alpha, which is more transmissible than wild-type (WT). The E484K mutation occurred in the receptor-binding domain, which is also responsible for the increased affinity to hACE2.
- Gamma shares the N501Y mutation with Alpha and Beta; it also shares the K417N/T and E484K mutations in the spike protein with Beta. Gamma exhibits increased transmissibility and risk of reinfection.
- Delta became the dominant variant by late 2021 in multiple countries since first detected in October 2020. In addition to the D614G, E484K and K417N mutations, L452R also increased infectivity. It is more contagious because of the faster replication rate and higher virus titers upon early infection.
- Omicron raises serious concerns as it harbors numerous mutations in the spike protein. It shares important mutations, namely, N501Y, K417N, P681H and E484K/A, in the spike protein with other four VOCs, which have been demonstrated to increase the infection and transmission rates.

**Neutralization heterogeneity of VOCs to sera elicited by vaccines or previous infections**

- Mutations in the spike proteins of VOCs might limit antibody-mediated neutralization because the design of major vaccines is based on the spike protein sequence of WT.
- Alpha demonstrated little resistance to antibodies elicited from convalescent and vaccine sera.
- Beta is more worrisome as it showed varying degrees of resistance to convalescent serum and vaccine-elicited antibodies.
- The neutralization activity of antibodies from convalescent sera and vaccinated individuals to Gamma was also reduced, although it is modest as compared with Beta.
- Vaccines remain protective against Delta, although multiple vaccines seemed to exhibit reduced effectiveness against the variant.
- The waning immunity may be responsible for the declined effectiveness and increased risk of reinfection.
- Booster vaccination is needed to enhance the waning immunity and prevent virus or breakthrough infection since the neutralization antibody titers decreased 6 months after receiving the second dose of the vaccines.
